# Syndrome congénital d´obstruction des voies aériennes supérieures (syndrome du CHAOS): à propos d’un cas

**DOI:** 10.11604/pamj.2021.38.1.27283

**Published:** 2021-01-01

**Authors:** Kamal El Moussaoui, Aziz Slaoui, Aziz Baidada, Aicha Kharabch

**Affiliations:** 1 Département de Gynécologie Obstétrique, Maternité Souissi, CHU Ibn Sina, Rabat, Maroc

**Keywords:** Syndrome du CHAOS, obstruction, voies aériennes supérieures, congénital, syndrome de Fraser, diagnostic prénatal, *case report*, CHAOS, congenital obstruction of upper airways, Fraser syndrome, prenatal diagnosis, case report

## Abstract

Le syndrome congénital d'obstruction des voies aériennes supérieures est une malformation rare qui est mortelle pour le nouveau-né après la naissance. Elle est définie comme une obstruction complète ou presque complète des voies aériennes supérieures. L'incidence réelle de la CHAOS est inconnue. Nous rapportons le cas d´une patiente de 24 ans, primipare sans antécédents médicaux ou chirurgicaux dont l´échographie de premier trimestre était sans anomalies. Au cours du deuxième trimestre l´examen échographique a montré un important œdème sous-cutané, avec un poumon hyperéchogène et hypertrophié, un cœur hypoplasique et une ascite importante associée à une oligoamnios sévères. Après avoir écarté les causes les plus fréquentes: l'iso-immunisation (Coombs indirecte négatifs), les infections (sérologies négatives) sans pouvoir disposer d'un caryotype, en raison du refus de la patiente, les résultats de l'échographie nous ont fait réfléchir à la possibilité d'un hydro-foetal secondaire au syndrome CHAOS, étant l'imagerie pathognomonique de l'hypertrophie pulmonaire avec inversion ou convexité des diaphragmes. Une IRM a été demandée montrant un probable syndrome CHAOS associé à d'autres malformations: atrésie laryngée, microphtalmie avec hypertélorisme et déviation de la cloison nasale avec absence de visualisation du tissu thymique, non identification claire de la vessie, absence de rein droit et un rein gauche hypoplasique. L´évolution de la grossesse a été marqué par la survenue À la 24^e^ semaine d´aménorrhée un accouchement prématuré qui a donné la naissance d´un nouveau-né avec un poids de naissance de 1475g, polymalformés présentant une ambiguïté sexuelle, avec un abdomen distendu et une polydactylie. Le nouveau-né est décédé à 3 min du post partum. Un caryotype était réalisé montrant un profil 46XX.

## Abstract (en)

Congenital upper airway obstruction syndrome is a rare malformation that can be fatal to a newborn baby. It is defined as a complete or almost complete obstruction of the upper airways. CHAOS rate is unknown. We here report the case of a 24-year-old primiparous patient with no medical or surgical history. First-trimester ultrasound didn’t show any abnormality. During the second trimester, ultrasonography showed extensive subcutaneous edema, hypertrophied lung with hyperechoic appearance, hypoplastic heart and large-volume ascites associated with severe oligoamnios. After having excluded the most common causes, including isoimmunization (indirect negative Coombs), infections (negative serologies) kariotiping was not performed because the patient refused it. The results of ultrasonography suggested fetal hydrops secondary to CHAOS syndrome, because of pathognomonic signs of pulmonary hypertrophy with inverted or convex diaphragm. MRI was requested; it suggested CHAOS syndrome associated with other malformations: laryngeal atresia, microphthalmia with hypertelorism and deviation of the nasal septum with lack of visualization of the thymic tissue, no clear identification of the bladder, absence of right kidney and hypoplastic left kidney. The outcome of pregnancy was marked by premature labour at the 24^th^ week of amenorrhea. The baby had a birth weight of 1475g, polimalformations such as sexual ambiguity, distended abdomen and polydactyly . The newborn died 3 min after birth. Karyotyping was performed which showed 46XX.

## Introduction

Le Syndrome congénital d'obstruction des voies aériennes supérieures (CHAOS) est défini comme une obstruction complète ou partielle des voies aériennes supérieures du fœtus. Cet état clinique a été introduit par Hedrick à la fin des années 1900 [1]. CHAOS est généralement causée par une atrésie ou une sténose du larynx ou trachée. L'incidence réelle de la CHAOS est inconnue. Si le syndrome n'est pas reconnu pendant la période prénatale période, elle se traduit généralement par une mort périnatale ou un décès de courte durée après l'accouchement [2]. Heureusement, il est possible de reconnaître davantage de cas in utero de nos jours, car il existe d'importantes améliorations de l'imagerie prénatale. Élargissement bilatéral, poumons hyperéchogènes, voies respiratoires dilatées et aplaties ou le diaphragme inversé est l'instrument d'échographie prénatale typique des résultats. L'ascite fœtale et l'anasarque non immunitaire peuvent être également associés à l'état clinique [3]. En raison des options de gestion récemment décrites, la définition prénatale d'obstruction des voies respiratoires du fœtus est l'espoir d'une issue néonatale [4].

## Patient et observation

**Figure 1:**
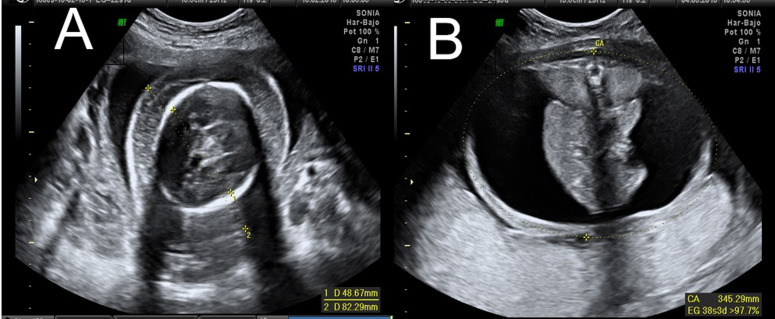
(A) biométrie de la calotte crânienne avec un œdème important; (B) ascite qui déforme la cavité avec une mauvaise visualisation de la chambre gastrique

**Figure 2:**
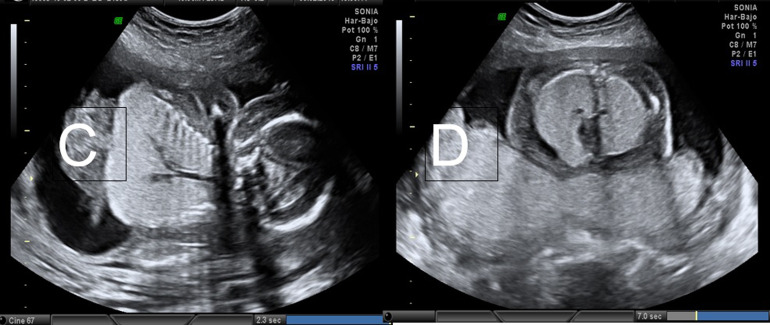
(C) trachée à un diamètre plus grand que les voies de sortie et les deux bronches principales dilatées; (D) cœur normal, ressemblant à une fausse hypoplasie, au détriment d'un volume pulmonaire accru

Patiente de 24 ans, primipare, sans antécédents médicaux ou chirurgicaux intéressants. L´échographie de dépistage à 12 semaines +2 jours était sans anomalie, avec un risque combiné de syndrome de Down et d'Edwards faible (<1/10000). L'échographie morphologique du deuxième trimestre (19 semaines +2 jours) a montré un œdème sous-cutané important, des poumons hyperéchogènes et hypertrophiés, un cœur hypoplasique et une ascite importante avec un oligoamnios sévères. Une nouvelle échographie de contrôle à 20 semaines a mis en évidence les résultats suivants: une biométrie de périmètre crânienne et une longueur du fémur normales pour un fœtus de 20 semaines d’aménorrhée. Cependant, la circonférence abdominale (254 mm) correspondait à un fœtus de 29 semaines d’aménorrhée, en rapport à un hydrops fœtal généralisé (Figure 1, Figure 2).

Au niveau céphalique, on a trouvé: une mauvaise visualisation du massif faciale en raison d'un œdème intense. Les orbites étaient marquées par une microphtalmie. Le nez et la bouche étaient sans anomalies évidentes. Au niveau abdominal, on a trouvé: la présence d'ascite qui déforme la cavité avec une mauvaise visualisation de la poche gastrique. Le rein gauche et le rein droit n'ont pas été visualisés, ils étaient petits et hypoplasiques. On a visualisé des doutes sur la rétraction de la vessie. Au niveau thoracique: le cœur présentait une anatomie normale, avec un aspect subjectif de fausse hypoplasie, au détriment de l'augmentation du volume des poumons. Les deux poumons étaient allongés, hypertrophiés et hyperéchogènes de façon homogène. De plus, la protubérance des deux diaphragmes était vers l'abdomen, avec une image inversée de ceux-ci, une image anormalement convexe, est frappante. La trachée avait un diamètre plus important que les voies de sortie et les deux bronches principales étaient pathologiquement dilatées. Une IRM a été demandée à 21^e^ semaine d’aménorrhée, qui était en faveur d´un syndrome CHAOS associé à d'autres malformations (possible syndrome de Fraser) à savoir la présence d´une atrésie laryngée, d´une Ascite et d´un hydrops foetal massif, d´une microphtalmie avec hypertélorisme et déviation de la cloison nasale, une absence de visualisation du tissu thymique, la présence d´une petite poche gastrique et une vessie non identifiable, absence de rein droit et un rein gauche hypoplasique.

**Figure 3:**
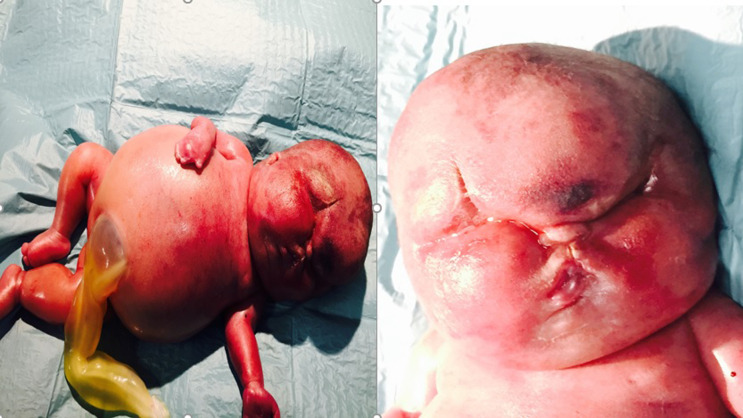
post-partum du nouveau-né atteint du syndrome CHAOS, montrant les différentes malformations

Un hydrops foetal a été considéré comme le diagnostic initial. Après avoir écarté les causes les plus fréquentes: isoimmunisation (Coombs indirect négatif), infections (sérologies négatives) sans pouvoir avoir un caryotype dû au rejet de la patiente, les résultats des ultrasons nous ont fait réfléchir à la possibilité d'un hydroscope fœtal secondaire au syndrome CHAOS, étant pathognomonique l'image d'une hypertrophie pulmonaire avec inversion ou convexité des diaphragmes. Lors des contrôles échographiques ultérieurs (23 semaines d’aménorrhée), la biométrie correspondait à 33 semaines d’aménorrhée, avec un grand œdème sous-cutané, un oligoamnios sévère et une ascite massive. L´évolution de la grossesse a été marquée par la survenue À la 24^e^ semaine d´aménorrhée un accouchement prématuré qui a donné la naissance d´un nouveau-né avec un poids de naissance de 1475g , polymalformés (Figure 3), présentant une ambiguïté sexuelle, avec un abdomen distendu et une polydactylie. Le nouveau-né est décédé à 3 min du post partum. Un caryotype était réalisé montrant un profil 46XX.

## Discussion

Le syndrome d'obstruction des voies aériennes supérieures est une malformation rare qui est mortelle pour le nouveau-né après la naissance. Elle est définie comme une obstruction complète ou presque complète des voies aériennes supérieures, qui peut être causée par diverses étiologies: atrésie laryngée, atrésie trachéale ou kystes ou “tissus” laryngés. L'atrésie laryngée peut être à son tour classée en: atrésie laryngée complète (type I), obstruction sous-glottique (type II) ou obstruction glottique (type III). Rossi, en 1826, a été le premier à décrire cette malformation [5-6]; depuis lors, les références bibliographiques sur ce syndrome sont rares, et encore moins les références aux cas correspondant à un diagnostic prénatal du syndrome. Entre 1987 et 1994, nous avons trouvé 16 cas décrits dans sa forme prénatale, dont un seul a survécu après une trachéotomie d'urgence lors de l'accouchement [7].

En revanche, de 1995 à aujourd´hui [5-8], nous avons trouvé 14 cas, dont 5 interruptions de grossesse (ILE) et 9 ont survécu, bien que seuls 8 aient présenté une survie à long terme. Ces 2 périodes ont permis de différencier 2 phases dans la connaissance du syndrome CHAOS. La première définit les critères utiles au diagnostic, sur la base des résultats des ultrasons concernant les changements structurels secondaires à l'atrésie. La deuxième phase décrit les traitements possibles et discute de leur gestion, et affine également le diagnostic en visualisant le processus obstructif. Selon la littérature publiée, EXIT (EXutero intrapartum treatment) pourrait être un outil important dans la gestion périnatale des cas où il y a de fortes suspicions d'obstruction des voies aériennes. Le premier cas traité avec succès a été décrit par DeCou *et al*. [9], mais il n'a pas été accompagné d'une survie à long terme. Cependant, Crombleholme *et al*. ont réussi en 2000.

Aujourd'hui, Kohl *et al*. [10] sont allés plus loin en réussissant à canaliser la zone obstruée dans les voies aériennes à l'aide de la fœtoscopie. On observe qu'après l'exécution de la technique, les signes échographiques secondaires à l'obstruction reviennent et la grossesse se termine avec succès. En outre, nous devons mentionner que dans ce cas, le fœtus présentait le syndrome de Fraser, non diagnostiqué par échographie. Cela nous fait penser qu'il est important de connaître et d'exclure les associations fréquentes des CHAOS avec d'autres conditions morphologiques et génétiques. Il faut plus d'expérience pour sélectionner correctement les patients atteints du syndrome CHAOS et sur la technique elle-même, en pesant les avantages et les risques de la technique. En outre, la possibilité de canaliser avec succès la zone obstruée doit être confirmée, en tenant compte de la difficulté que peut présenter la canalisation d'une zone fortement altérée et sténosée.

## Conclusion

L'obstruction de la voie aérienne supérieure correspond à une malformation mineure fréquente, mortelle intra-utérine ou dans la période post-partum immédiate. Il est important d'informer la patiente sur cette pathologie, son pronostic et évolution pour la prise de décision.
